# Lactate Stimulates a Potential for Hypertrophy and Regeneration of Mouse Skeletal Muscle

**DOI:** 10.3390/nu11040869

**Published:** 2019-04-17

**Authors:** Yoshitaka Ohno, Koki Ando, Takafumi Ito, Yohei Suda, Yuki Matsui, Akiko Oyama, Hikari Kaneko, Shingo Yokoyama, Tatsuro Egawa, Katsumasa Goto

**Affiliations:** 1 Laboratory of Physiology, School of Health Sciences, Toyohashi SOZO University, Toyohashi 440-8511, Japan; yohno@sozo.ac.jp (Y.O.); r1482701@sc.sozo.ac.jp (K.A.); r1482703@sc.sozo.ac.jp (T.I.); r1482716@sc.sozo.ac.jp (Y.S.); r1482724@sc.sozo.ac.jp (Y.M.); r1382605@sc.sozo.ac.jp (A.O.); r1382606@sc.sozo.ac.jp (H.K.); s-yokoyama@sozo.ac.jp (S.Y.); 2 Laboratory of Sports and Exercise Medicine, Graduate School of Human and Environmental Studies, Kyoto University, Kyoto 606-8501, Japan; egawa.tatsuro.4u@kyoto-u.ac.jp; 3 Department of Physiology, Graduate School of Health Sciences, Toyohashi SOZO University, Toyohashi 440-8511, Japan

**Keywords:** lactate, skeletal muscle, hypertrophy, regeneration, muscle satellite cell

## Abstract

The effects of lactate on muscle mass and regeneration were investigated using mouse skeletal muscle tissue and cultured C2C12 cells. Male C57BL/6J mice were randomly divided into (1) control, (2) lactate (1 mol/L in distilled water, 8.9 mL/g body weight)-administered, (3) cardio toxin (CTX)-injected (CX), and (4) lactate-administered after CTX-injection (LX) groups. CTX was injected into right tibialis anterior (TA) muscle before the oral administration of sodium lactate (five days/week for two weeks) to the mice. Oral lactate administration increased the muscle weight and fiber cross-sectional area, and the population of Pax7-positive nuclei in mouse TA skeletal muscle. Oral administration of lactate also facilitated the recovery process of CTX-associated injured mouse TA muscle mass accompanied with a transient increase in the population of Pax7-positive nuclei. Mouse myoblast-derived C2C12 cells were differentiated for five days to form myotubes with or without lactate administration. C2C12 myotube formation with an increase in protein content, fiber diameter, length, and myo-nuclei was stimulated by lactate. These observations suggest that lactate may be a potential molecule to stimulate muscle hypertrophy and regeneration of mouse skeletal muscle via the activation of muscle satellite cells.

## 1. Introduction

Muscle satellite cells are known as skeletal muscle-specific stem cells that reside between the basal lamina and sarcolemma of mature myo-fibers [1]. Muscle satellite cells, which express the paired box transcription factor 7 (Pax7), are normally quiescent but become activated in response to exercise or injury [2,3,4]. Activated muscle satellite cells proliferate and undergo differentiation into myoblasts. Then the myoblasts differentiate and fuse into preexisting myofibers or fuse to form new myofibers, which result in skeletal muscle hypertrophy or regeneration [5,6,7].

Muscle satellite cells are considered to play a crucial role in exercise-associated muscle hypertrophy in human skeletal muscles [6,8] even though the studies using rodent models indicated that satellite cells may not contribute to exercise-associated hypertrophy of skeletal muscle [9,10]. Furthermore, exercise-associated stimuli, such as mechanical and heat stresses, are proposed to be potential stimuli to activate the regenerative process of injured skeletal muscle [11,12,13]. However, the mechanism of exercise-induced hypertrophy and regeneration of skeletal muscle is not fully elucidated.

Recent studies demonstrate that the number of biologically active molecules, so-called myokines, are released from resting as well as contracting skeletal muscle cells [14]. It is generally accepted that intensive exercise induces the release of lactate from contracting skeletal muscle. Extracellular lactate is re-uptaken by skeletal muscle to utilize it for an energy source [15,16]. On the other hand, the previous study using C2C12 skeletal muscle cells showed that a high level of extracellular lactate changed the expression of follistatin and myostatin [17], which regulate the proliferation of muscle satellite cells [18]. Furthermore, we recently demonstrated extracellular lactate-associated C2C12 myotube hypertrophy by activating the anabolic intracellular signals, such as p42/44 extracellular signal-regulated kinase-1/2 (ERK1/2) pathway [19], which stimulates muscle cell proliferation and differentiation [20,21,22]. Judging from published results, we hypothesize that increasing extracellular lactate level, which is generally induced by intensive exercise, may induce muscle hypertrophy as well as regeneration of injured skeletal muscle by activating muscle satellite cells.

In the present study, we investigated the effects of oral lactate administration on hypertrophy and regeneration in mouse skeletal muscle. Since previous studies have reported that an increase of satellite cells, which is caused by extracellular stimuli including electrical and heat stimulation [11,23], facilitated muscle regeneration, we evaluated the population of satellite cells following lactate administration. The effects of lactate on the formation of myotubes were also investigated by using cultured C2C12 cells.

## 2. Materials and Methods

### 2.1. Animal Experiments

All animal experimental procedures were conducted in accordance with the Guide for the Care and Use of Laboratory Animals, as adopted and promulgated by the National Institutes of Health (Bethesda, MD, USA). The Animal Use Committee of Toyohashi SOZO University (A2016003, A2017002) approved the procedures of animal experiments in this study. Male C57BL/6J mice aged 8-week old were used. To investigate the effects of lactate on skeletal muscle hypertrophy or regeneration, mice were randomly divided into control (C) and lactate-administered (L) groups (*n* = 24), or cardio-toxin (CTX)-injected (CX) and lactate-administered after CTX injection (LX) groups (*n* = 28). All mice were housed in a clean room controlled at approximately 23 °C with a 12/12 h light-dark cycle. Solid diet and water were provided ad libitum.

Muscle injury-regeneration cycle was induced by injecting 0.1 mL cardiotoxin (CTX, 10 μmol/L in physiological saline, Sigma-Aldrich, St. Louis, MO, USA) of Naja naja atra venom into right tibialis anterior (TA) muscles in the CX and LX groups. After epilation of right hind limb with a commercial hair remover for human, injection of CTX into right TA muscle was performed using a 27-gauge needle. During this procedure, all mice were under anesthesia with intraperitoneal injection of sodium pentobarbital (50 mg/kg) [23].

In the L and LX group, sodium lactate (1 mol/L in distilled water, 8.9 mL/g body weight, Otsuka Pharmaceutical Factory, Inc., Naruto, Tokushima, Japan) was administered to the mice by using an oral sonde 5 days a week for 2 weeks after CTX injection. The dose (or amount) of lactate was selected considering the previous rat study [18]. The same volume of ultrapure water was administered to the C and CX groups. In a pilot study, the changes of blood lactate concentration, which was collected from the tail vein of mice after oral lactate administration, were evaluated using the Lactate Pro2 blood lactate test meter (ARKRAY, Inc., Kyoto, Japan). Before the administration of lactate, the blood lactate concentration of mice (*n* = 7) was 2.9 ± 0.2 mmol/L (Table 1). Two hours after the lactate administration, blood lactate level significantly increased up to 4.1 ± 0.3 mmol/L. Similar to this observation, the oral administration of lactic acid to rats led to a rise in the blood level [24].

Mice were sacrificed by cervical dislocation under anesthesia with intraperitoneal injection of sodium pentobarbital (50 mg/kg) 1 and 2 weeks after CTX injection. Immediately after the sacrification, right TA muscle was excised. Dissected TA muscles were rapidly weighed and frozen in isopentane cooled in liquid nitrogen. All samples were then stored at −80 °C until analyses.

Serial transverse cryo-sections (8-μm thick) of the samples were cut at −20 °C and immediately mounted onto glass slides. Sections were stained to analyze the cross-sectional area (fiber CSA) of muscle fibers by hematoxylin and eosin (H&E), and the profiles of Pax7-positive nuclei by a standard immuno-histochemical technique [23]. Monoclonal anti-Pax7 antibody (Developmental Studies Hybridoma Bank, Iowa, IA, USA) was used for the detection of muscle satellite cells [3]. The sections were fixed in 4% paraformaldehyde, and were then post-fixed in ice-cooled methanol. After blocking by using 1% Roche blocking reagent (Roche Diagnostic, Penzberg, Germany), sections were incubated with the primary antibodies for Pax7 and rabbit polyclonal anti-laminin (Z0097, DakoCytomation, Glostrup, Denmark). Following an incubation period at 4 °C, sections were incubated with secondary antibodies for Cy3-conjugated anti-mouse IgG (Jackson Immuno Research, West Grove, PA, USA) and with fluorescein isothiocyanate-conjugated anti-rabbit IgG (Sigma-Aldrich) at room temperature. Nuclear counterstaining was performed in a solution of 4′,6-diamidino-2-phenylindole dihydrochloride (DAPI, Sigma-Aldrich). The number of Pax7-positive nuclei located within the laminin-positive basal membrane per muscle fiber (approximately 250 fibers) from each muscle was calculated. Using H&E stained sections, mean fiber CSA was measured from approximately 250 fibers of each muscle using the National Institutes of Health Image J 1.38X (NIH, Bethesda, MD, USA) software for Windows.

### 2.2. Cell Culture Experiments

Mouse myoblast-derived C2C12 cells (6 × 10^4^ cells/well) were cultured on 6-well culture plates, coated with type I collagen (Biocoat, Corning, NY, USA). Cells were maintained in 2 mL of growth medium that consisted of Dulbecco’s modified Eagle’s medium (DMEM, Thermo Fisher Scientific, Yokohama, Japan) supplemented with 10% heat-inactivated fetal bovine serum containing high glucose (4.5 g/L glucose, 4.0 mM L-glutamine, without sodium pyruvate) for proliferation. During the third day of the proliferation phase (at ~80% confluence), the culture medium was then changed to the same amount of differentiation medium, which consisted of DMEM supplemented with 2% heat-inactivated horse serum containing low glucose (1.0 g/L glucose, 4.0 mM L-glutamine, and 110 mg/L sodium pyruvate) for differentiation, as was described previously [19]. Every 2 days, cells were replenished with fresh differentiation medium and cultures were maintained for 5 days. All cells were maintained at 37 °C, under a humidified atmosphere with 5% CO_2_ and 95% air.

Sodium lactate was administered into the conditioned medium throughout the differentiation phase. The concentration of lactate was set at 20 mM in the conditioned culture medium, which took into account previous studies using skeletal muscle cells [19,25]. Ultrapure water alone was added to the conditioning medium for the control group (*n* = 6 well in each group).

The images of myotubes at the 5th day of the differentiation phase were visualized at x40 magnification using a calibrated color imaging camera (DP12, Olympus, Tokyo, Japan) set up to a phase contrast light microscope (CK40, Olympus). In order to measure the myotube diameter, we used a modified method of a previous study [26]. We first randomly selected fields of view from 6 wells of each condition. Using Image J, the diameters of at least 100 myotubes in each well were measured at three randomly selected portions taken along the length of the myotube. Then, the average diameter of a myotube was calculated as the mean of three measurements. The myotubes were also used for the evaluation of myotube length and the myo-nuclei number as described below. Cells were fixed with 4% paraformaldehyde. After blocking, cells were incubated with the primary antibodies for skeletal myosin (M4276, Sigma-Aldrich). Cells were then incubated with the secondary antibodies for Cy3-conjugated anti-mouse IgG. Then nuclei were counterstained with DAPI. Since a muscle cell containing 3 or more nuclei was considered to be a myotube [27], the myotube length and myonuclei number in differentiated myotubes (>2 myonuclei) were measured using Image J.

In addition, the myotubes at the 5th day of differentiation were used to analyze muscular protein content as described below. In order to extract protein from C2C12 cells, the cells were lysed in a cell lysis reagent (CelLytic^TM^-M, Sigma-Aldrich), in accordance with the previously reported method [19]. The cells in each well were rinsed twice in 1 mL of ice-cooled phosphate-buffered saline. The cells of each well were then scraped into 0.3 mL of cell lysis reagent on ice. The cell lysate was sonicated and centrifuged at 20,000 *g* at 4 °C for 10 min. The supernatant was collected for the analysis of protein content. Protein content in the supernatant was determined using the Bradford technique (protein Assay kit, Bio-Rad, Hercules, CA, USA) and bovine serum albumin (Sigma-Aldrich) as the standard.

### 2.3. Animal Experiments

All values were expressed as means ± SEM. In animal experiments, the statistically significant level of blood lactate concentration was analyzed using one-way analysis of variance (ANOVA) followed by the Tukey-Kramer test. Other significant levels were tested using a two-way (lactate administration and time) ANOVA for multiple comparisons followed by the Tukey-Kramer test. When a significant interaction between main factors was observed, one-way ANOVA followed by the Tukey-Kramer test was performed. Statistically significant levels in cell experiments were evaluated using unpaired Student’s *t*-test following F-test. The significance level was accepted at *p* < 0.05.

## 3. Results

### 3.1. Effect of Lactate on Skeletal Muscle Hypertrophy

**Figure 1:**
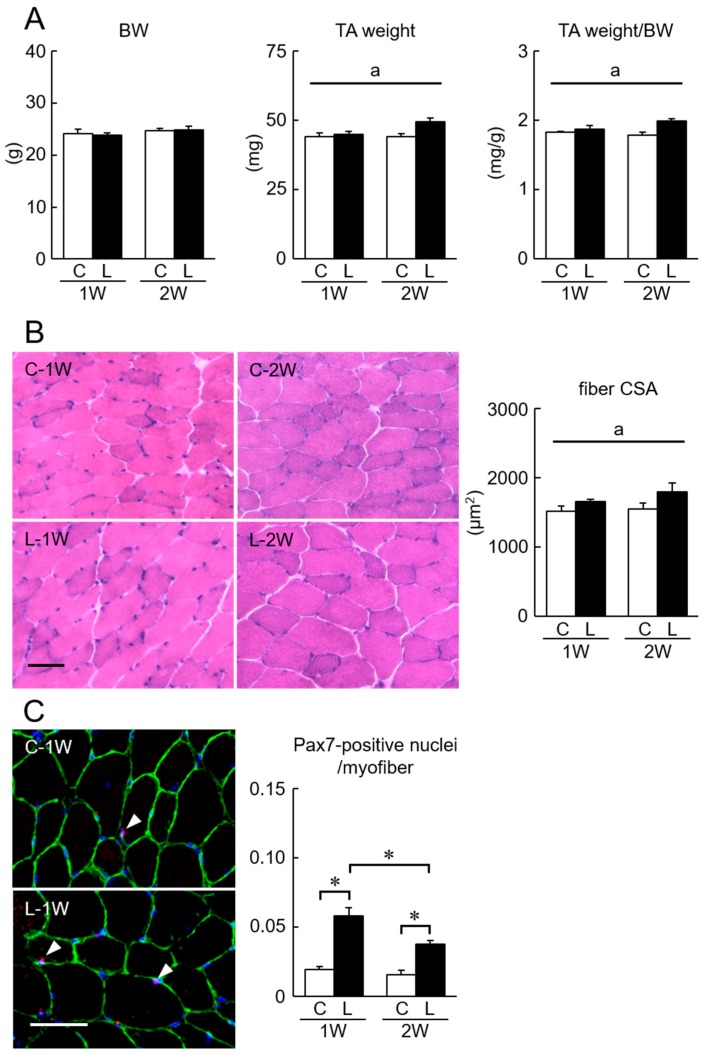
(**A**) Effects of oral lactate administration on body weight (BW) and tibialis anterior (TA) weight in mice. Effect of oral lactate administration on the TA muscle fiber cross-sectional area (CSA) (**B**) and the number of Pax7-positive nuclei per muscle fiber (**C**). Representative images of histochemical and immuno-histochemical staining in TA muscle are shown. Arrowheads indicate the Pax7-positive nuclei. Scale bar = 50 µm. TA weight/BW: relative TA weight to BW. C: control group. L: lactate-administered group. 1W and 2W: 1 and 2 weeks of lactate administration, respectively. Values are means ± SEM. *n* = 6 per group. a: Significant main effect of lactate, *p* < 0.05. *: *p* < 0.05.

The body weight, TA muscle weight, and fiber CSA in response to oral lactate administration were shown in Figure 1. There was no significant difference in body weight between groups. A significant increase in the absolute TA muscle weight and the muscle weight relative to body weight was observed in the lactate-administered L group (Figure 1A, *p* < 0.05). Similarly, the fiber CSA in the L group was significantly larger than that in the C group (Figure 1B, *p* < 0.05).

There was no significant change in the population number of Pax7-positive nuclei a fiber of TA muscle in the C group during the experimental period. In the L group, the population of Pax7-positive nuclei was significantly increased (200% and 138% at 1 and 2 weeks after lactate administration, respectively), compared with that in the C group (Figure 1C, *p* < 0.05).

### 3.2. Effect of Lactate on Skeletal Muscle Regeneration

**Figure 2:**
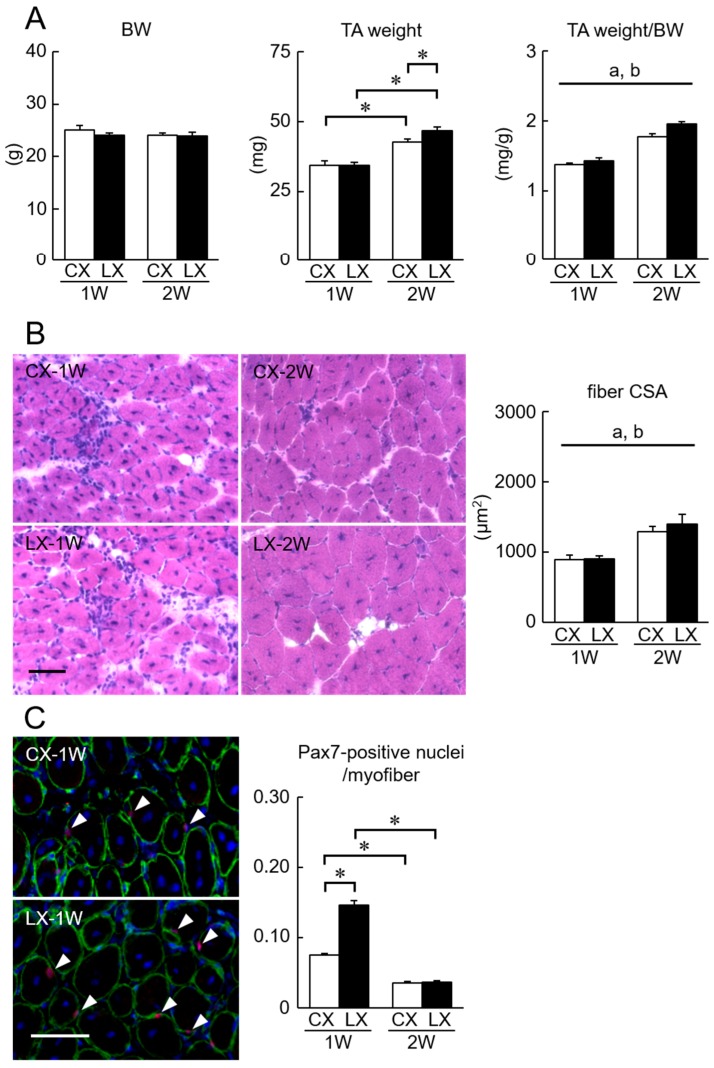
(**A**) Effects of oral lactate administration on BW and TA weight in cardio-toxin (CTX)-injected mice. Effect of oral lactate administration on CTX-injected TA muscle fiber CSA (**B**) and the number of Pax7-positive nuclei per muscle fiber (**C**). Representative images of histochemical and immuno-histochemical staining in CTX-injected TA muscle are shown. Arrowheads indicate the Pax7-positive nuclei. Scale bar = 50 µm. CX: CTX-injected group. LX: lactate-administered after the CTX-injection group. 1W and 2W: 1 and 2 weeks after CTX injection, respectively. See Figure 1 for other abbreviations. Values are means ± SEM. *n* = 7 per group. a and b: Significant main effect of lactate and time, respectively. *p* < 0.05. *: *p* < 0.05.

Many regenerating fibers with centrally located nuclei in CTX-injected TA muscle were observed 1 and 2 weeks after the injection (Figure 2B). There was no significant change in body weight of not only CX but also LX groups during the experimental period (Figure 2A). Both TA muscle weight (24% and 29% of absolute and relative levels, respectively) and fiber CSA (44%) 2 weeks after CTX injection increased, compared with those 1 week after the injection. Two weeks after CTX injection, lactate administration-associated increase in the absolute TA muscle weight was observed (*p* < 0.05). A significant main effect of lactate administration was observed in the relative muscle weight as well as fiber CSA (*p* < 0.05).

One week after CTX injection, the population of Pax7-positive nuclei was increased (0.08/myofiber), compared with that in the C group (0.02/myofiber). Furthermore, the additional increase in the population of Pax7-positive nuclei was observed in the lactate-administered LX group, when compared with the CX group, 1 week after CTX injection (*p* < 0.05).

### 3.3. Effects of Lactate on Myotube Formation

**Figure 3:**
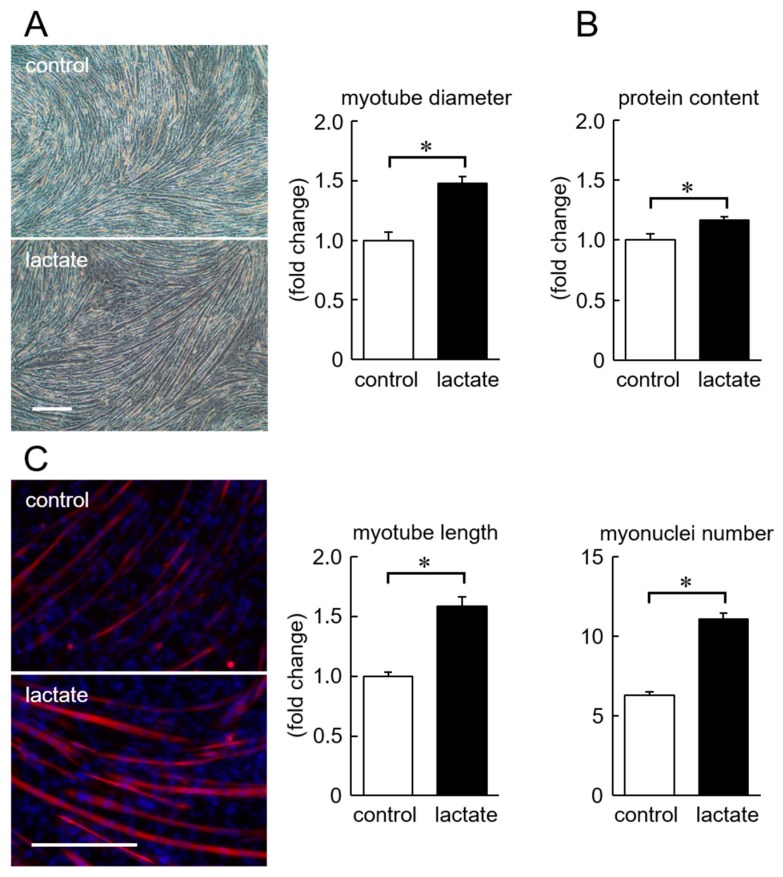
Myo-tube diameter (**A**), muscular protein content (**B**), myo-tube length, and myonuclei number (**C**) of C2C12 cells in response to lactate. Scale bar = 300 µm. Values are means ± SEM. *n* = 6 wells per group. *: *p* < 0.05.

We examined the effects of lactate on myotube formation of C2C12 cells. The typical images of myotubes, on the 5th day of differentiation, with or without lactate in the medium were shown in Figure 3. In the lactate-administered cells, myotubes have a wider diameter, longer length, and more myonuclei compared with the control. A significant increase in the myotube diameter and muscular protein content was induced by lactate (Figure 3A,B, *p* < 0.05). Lactate administration also increased myotube length as well as myo-nuclei number (Figure 3C, *p* < 0.05).

## 4. Discussion

The present study demonstrated that oral lactate administration induced muscle hypertrophy accompanied with an increase of Pax7-positive nuclei in mouse TA muscle. In injured TA muscle, the increase of muscle mass and Pax7-positive nuclei population was stimulated by lactate administration. Furthermore, lactate-induced myotube formation including higher protein content, wider diameter, longer length, and more myo-nuclei were observed in C2C12 cells.

### 4.1. Muscle Hypertrophy

In the present study, oral lactate administration increased TA muscle weight and fiber CSA in mice. This is the first report showing the effect of lactate on skeletal muscle mass in animals. Furthermore, the population of Pax7-positive nuclei in TA muscle was increased by lactate administration. Since the previous study using C2C12 skeletal muscle cells reported that extracellular lactate increased follistatin and decreased myostatin expressions involved in the proliferation of satellite cells [17,18], oral lactate administration-associated increase of blood lactate concentration may enhance the proliferation of muscle satellite cells. Recently, we demonstrated an extracellular lactate-associated increase in the diameter of C2C12 myo-tubes [19]. These observations suggest that lactate administration, which could increase blood lactate levels, stimulated the hypertrophy of skeletal muscle with the activation of muscle satellite cells. Training with a blood flow restriction, which is exercise with vascular occlusion, is known to increase muscle size [28,29] and the blood lactate level [29,30] in humans. Therefore, there is a possibility that the blood lactate level may contribute to muscle size following occlusion training.

### 4.2. Muscle Regeneration

In the present study, increase in muscle weight and fiber CSA in CTX-injected TA muscle was observed during a 2-week experimental period. In addition, the population of Pax7-positive nuclei was increased by CTX injection. These phenomena are consistent with the previously reported data in mice [23]. Larger muscle mass and Pax7-positive nuclei population were also observed in lactate-administered mouse, which suggests that lactate stimulates the regenerative potential of injured skeletal muscle by activating muscle satellite cells.

### 4.3. Myotube Formation

In the present study, lactate increased the myotube diameter and protein content in C2C12 cells. These results are supported by the previous report that lactate caused the activation of anabolic signals for hypertrophy and myogenesis in skeletal muscle cells [18]. Our previous data also showed that extracellular lactate increased the diameter of C2C12 myotubes in a dose-dependent manner [19]. In addition, the present study demonstrated that extracellular lactate caused the extension of the myotube length and the increase of the myonuclei number. Therefore, it was suggested that lactate may stimulate the fusion of myoblasts, which results in myotube formation. This contributes to muscle hypertrophy and regeneration [5,6,7].

It has been a debatable argument that various metabolites, including lactate, may be involved in exercise-associated skeletal muscle hypertrophy [31]. In the present study, cell culture experiments demonstrated lactate induces an increase in muscle mass even though no myotube contraction is observed. On the other hand, lactate may also stimulate the hypertrophic effects of physical activity on skeletal muscle, since mice moved freely immediately after the administration of lactate in the present study. Additional results are needed to elucidate the difference and interaction between lactate and muscle contraction in skeletal muscle hypertrophy.

## 5. Conclusions

The present study demonstrated oral lactate administration-associated hypertrophy and regeneration of mouse skeletal muscle. Extracellular lactate might contribute to the regulation of skeletal muscle plasticity.

## Figures and Tables

**Table 1:** Blood lactate concentration of mice following orally administered sodium lactate.

	Pre	2 h	6 h	24 h
Concentration, mmol/L	2.9 ± 0.2	4.1 ± 0.3 ^†^	2.9 ± 0.3 ^§^	3.0 ± 0.4

Pre: before oral lactate administration (base line); 2 h, 6 h, and 24 h: 2, 6, and 24 h after the lactate administration. Values are means ± SEM. *n* = 7. ^†^ and ^§^: *p* < 0.05 vs. Pre and 2 h, respectively.
